# Spectrum of BCR-ABL mutations in Azerbaijanian imatinib-resistant patients with chronic myeloid leukemia

**DOI:** 10.3389/pore.2023.1611518

**Published:** 2023-12-22

**Authors:** Aypara Hasanova, Chingiz Asadov, Nigar Karimova, Aytan Shirinova, Gunay Aliyeva, Zohra Alimirzoyeva

**Affiliations:** ^1^ Leukemogenesis Laboratory, Institute of Hematology and Transfusiology, Baku, Azerbaijan; ^2^ Hematopoiesis Department, Institute of Hematology and Transfusiology, Baku, Azerbaijan; ^3^ Human Genetics Laboratory, Biotechnology Department, Institute of Genetic Resources, Baku, Azerbaijan; ^4^ Hematology Department, Institute of Hematology and Transfusiology, Baku, Azerbaijan

**Keywords:** chronic myeloid leukemia, mutation, tyrosine kinase inhibitor resistance, kinase domain, BCR-ABL

## Abstract

**Objective:** BCR-ABL1 kinase domain (KD) mutations can lead to resistance to first- and second-generation tyrosine kinase inhibitors (TKIs) in chronic myeloid leukemia (CML). Here, we present the first report of the spectrum of mutations in the BCR-ABL1 KD of CML patients from Azerbaijan.

**Materials and methods:** Samples for mutation screening were obtained from patients experiencing resistance to first line TKIs or from patients in acceleration phase (AP) or blast crisis (BC) at the time of diagnosis. The cDNA region corresponding to BCR-ABL1 KD was sequenced by pyrosequencing method. The χ^2^ test was used to assess the association of categorical variables between mutation-positive and -negative groups. In addition, the Kaplan-Meier method was applied to generate survival curves.

**Results:** Eight different point mutations were identified in 22 (13.4%) out of 163 CML patients experiencing resistance to TKIs. The types of mutations detected were as follows: Contact binding site mutations 50% (11), SH2 domain mutations 27.4% (six), P-loop mutations 18.1% (four), and SH3 domain mutations accounting for 4.5% (one). The most common mutation was T315I, accounting for 5% (*n* = 8) of all patients. Significant association was identified between BCR-ABL1 mutations and additional chromosomal aberrations as well as between the mutations and disease phases (*p* < 0.05). Twelve out of 22 patients with BCR-ABL1 mutations and seven out of eight with T315I were in BC. Overall survival (OS) of the patients with BCR-ABL1 mutations was significantly lower comparing to the patients with no mutation (*p* < 0.05) and 8 patients with T315I mutation presented OS of 0%.

**Conclusion:** T315I was the most commonly identified BCR-ABL1 mutation in TKI-resistant CML patients of Azerbaijani origin, being associated with disease progression and poor OS.

## Introduction

Rearrangements involving the ABL1 gene at chromosome 9q and the BCR gene at 22q result in formation of the Philadelphia (Ph) chromosome, t(9; 22)(q34; q11), and are present in all cases of chronic myeloid leukemia (CML) and in some cases of acute lymphoblastic leukemia (Ph+ ALL). The BCR-ABL1 fusion gene, characteristic for these patients, encodes an oncoprotein that has an activated tyrosine kinase domain in the ABL region. This infamous protein promotes cell proliferation leading to loss of stromal adhesion and inhibits apoptosis by activating downstream pathways, including Janus kinase-signal transducer and activator of transcription (Jak-Stat) and Myc [1, 2].

Introduction and widespread clinical use of the tyrosine kinase inhibitor (TKI), imatinib in CML patients, has resulted in much better overall survival compared to interferon [3]. Furthermore, imatinib has reduced the need for allogeneic stem cell transplantation in these patients [4]. Despite the extraordinary success of imatinib for the treatment of CML, up to 40% of patients experience intolerance or resistance. The most common reason for treatment failure is the acquisition of mutations within the kinase domain (KD) of BCR-ABL1 that impair drug binding. Although more developed second-generation TKIs were designed to target most imatinib-resistant mutations, some mutations show resistance even to these new generation drugs (T315I) [5, 6]. Almost 25% of imatinib-resistant patients were reported to have more than one BCR-ABL1 KD mutation, and the presence of multiple mutations defines a poor-risk subgroup of chronic phase (CP) CML patients [7]. These patients have a higher likelihood of acquiring new resistant mutations and poor response to treatment with second-generation TKIs, irrespective of the resistance profile of the mutations [7].

Currently, TKIs applied to treat CML strongly improve the overall survival (OS) of patients [2, 8]. The majority of patients achieve a life expectancy close to that of the general population [9]. However, there are still some treatment-related problems present: 1) The disease still progresses to an acceleration phase (AP) or blast crisis (BC) following treatment with TKIs; 2) a number of patients are resistant to TKIs; and 3) patients cannot endure the side effects of TKIs [10].

Patients frequently develop primary or secondary resistance to TKIs, despite the high response rates (primary or intrinsic resistance is a lack of initial response, whereas secondary or acquired resistance (relapse) is a loss of an established response) [11]. According to the European LeukemiaNet (ELN) criteria, resistance is defined as failure of first-line TKI therapy: less than a complete hematologic response and/or no cytogenetic response (CyR; defined as Ph+ bone marrow metaphases >95%) at 3 months, BCR-ABL1 transcript levels above 10% and/or less than a partial CyR (PcyR; defined as ≤35% Ph+ metaphases) at 6 months, BCR-ABL1 transcript levels above 1% and/or less than a complete CyR (CCyR; defined as no Ph+ metaphases) at 12 months, or loss of a complete hematologic response or complete CyR or confirmed loss of MMR, mutations, or clonal chromosome abnormalities in Ph− cells at any subsequent time during therapy [1, 12]. According to the National Comprehensive Cancer Network (NCCN) guidelines, on the other hand, resistance is defined as the detection of BCR-ABL1/ABL1 transcript levels above 10% on the International Scale or failure to achieve PCyR at 3 months, or failure to achieve CCyR at 12 months or 18 months [13]. Several mechanisms have been associated with resistance, including BCR-ABL1-dependent and BCR-ABL1-independent mechanisms [14]. BCR-ABL1 KD mutations and BCR-ABL1 genomic amplification are well-known mechanisms of resistance to TKI therapy [15].

Mutation analysis is usually performed after patients experience TKI treatment resistance; the results of mutation analysis may guide the selection of subsequent TKIs [1, 12, 13, 16]. Imatinib resistance, regardless of aetiology, necessitates a change in treatment strategy. However, the choice of second-generation TKI should take into account ABL1 KD mutation status to ensure optimum efficiency of TKI. Imatinib resistance often results from ABL1 mutations in the P-loop (G250E or E255K/V) and in the ATP-binding site (F317L) or from the panresistant mutation T315I [17–20]. Only few mutations are known to cause clinical resistance to nilotinib (Y253H, E255K/V, and F359V/C/I) [21, 22] or dasatinib (V299L, T315A, F317L/I/V/C) [22–25] or both (T315I). Patients already having mutations have higher likelihood of developing new mutations [15, 22] and multiple mutations can be associated with poor prognosis [24, 26, 27]. BCR-ABL1 KD mutation analysis can be done either by direct Sanger sequencing or Pyrosequencing which has been largely used to-date [1, 16]. Although newer technologies with greater sensitivity are available, most of them are limited by their specificity and limited spectrum of mutations, next-generation sequencing (NGS), nevertheless, is an exception.

The aim of this study is thus to analyse the association of identified BCR-ABL1 KD mutations with resistance to first-line TKI imatinib.

## Methods

### Patients

In this study, 163 CML patients treated in the clinical department of the Scientific Research Institute of Hematology and Transfusion were analyzed retrospectively. At the time of the analysis, 119 patients were in the CP, 26 were in the AP, and 18 were in the BC phase. Phases of the disease were determined according to ELN recommendations [1]. Mutation screening for BCR-ABL1 KD was performed in CML patients who showed resistance to imatinib treatment and failed to achieve MMR after 12, 18, and 24 months. Data was collected from patients undergoing mutation screening during imatinib treatment. Mutation screening was performed in patients who showed resistance to the drug or progressed to AP or BC phase of the disease, as required. Patients were grouped according to BCR-ABL1 mutational status. The relationship between the clinical data of the patients and the mutation status was analyzed. The study was approved by institutional review board and consent was obtained from all patients included in the study.

### Real-time polymerase chain reaction (RT PCR)

Evaluation of p210 BCR/ABL1 chimeric oncogene expression was implemented in several steps: RNA isolation; Obtaining cDNA by reverse transcriptase reaction; Real-time PCR. RNA isolation was performed using QIAamp^®^ RNA Blood Mini Kit (QiaGen, Germany), Reverse Transcriptase RT^®^ Kit (QiaGen, Germany). Quantification of BCR-ABL1 p210, b2a2 or b3a2 transcripts in bone marrow or peripheral blood samples of CML patients was carried out by RT PCR on Rotor-Gene Q (QiaGen, Germany) device and BCR-ABL1 Mbcr IS-MMR Kit (QiaGen, Germany). The background level (LoB) equal to 0.0022 BCR-ABL Mbcr NCN. The limit of detection (LoD or analytical sensitivity) equal to 0.0069 BCR-ABL Mbcr NCN.

### Fluorescence *in situ* hybridization (FISH) method

FISH analysis was performed using the BCR/ABL(ABL1) Translocation, Dual Fusion DNA probe (Cytocell, United Kingdom) on metaphase chromosomes or interphase nuclei obtained by short-term culture. The cut-off value was 2.39% for bone marrow and 2.55% for peripheral blood. Preparations were examined using an Imager A2 microscope (Carl Zeiss), Isis software (Metasystems) and DAPI/TexasRed/FITC filters.

### Cytogenetic method

Metaphases obtained after culturing bone marrow (24 h) or peripheral blood samples (72 h) were stained by GTL-banding. Karyotyping of metaphases was performed using an Axio Imager A2 microscope (Carl Zeiss) and Ikaros software (Metasystems).

### BCR-ABL1 mutation analysis

These examinations were carried out with the support of ATQ Biotechnology Laboratory operating in Ankara, Türkiye. Pyrosequencing analysis is based on the principle of sequencing by synthesis. This method is a real-time quantitative sequence analysis based on the detection of pyrophosphates released during DNA synthesis. All tests were performed in peripheral blood. First, RNA was isolated from peripheral blood samples with the QIAamp^®^ RNA Blood Mini Kit (QiaGen, Germany). In the next step, RNA to cDNA was converted using RT^®^ Kit (QiaGen, Germany) to obtain cDNA. Then, two rounds of PCR were performed using the Pyromark PCR kit (QiaGen, Germany). After the PCR step, pyrosequencing analysis was performed on a Pyromark Q24 (QiaGen, Germany) device. The investigation of mutations resulting from single nucleotide variants (SNVs) was carried out through BCR-ABL1 pyrosequencing analysis, as outlined in Table 1.

**TABLE 1:** Mutations determined by BCR-ABL1 pyrosequencing analysis.

No	Sequencing primer	Amino acid change	Point mutation	Sequence
1	BCR-ABL 253 Forward	Y253H	(TAC>CAC)	1: T/CAC​GGG​G/AAG​GTG​TAC​GA
2		Y253F	(TAC>TTC)	
3		E255K	(GAG>AAG)	2: TA/TCG​GGG​A/TGG​TGT​ACG​A
4		E255V	(GAG>GTG)	
5	BCR-ABL 299 Reverse	V299L	(GTG>CTG)	AC/G/ACA​GGT​TAG​GGT​GTT​T
6		V299L	(GTG>TTG)	
7	BCR-ABL 315 Forward	T315A	(ACT>GCT)	A/GC/TTG​AGT/C/GTC/A/GAT​GAC​CT
8		T315I	(ACT>ATT)	
9		F317L	(TTC>TTA)	
10		F317L	(TTC>TTG)	
11		F317L	(TTC>CTC)	
12		F317V	(TTC>GTC)	
13	BCR-ABL 359 Forward	F359V	(TTC>GTC)	1: CT/GTCATCCACA
14		F359C	(TTC>TGC)	2: CTT/GCATCCACA

### Statistical analyses

To assess the associations between two groups of categorical variables, χ^2^ test was used. OS was calculated from the identification of the mutation until the last follow-up, death or the censor date. To generate survival curves, the Kaplan-Meier method was applied. *p* < 0.05 was considered to indicate a statistically significant difference.

## Results

### Patient characteristics

The clinical, hematologic, and epidemiologic characteristics were analyzed altogether during the diagnosis and mutation screening of the 163 patients included in this study (Table 2). Patients were stratified by BCR-ABL1 mutation status. No significant difference was observed between clinical characteristics of the two groups, except for gender and mean age: male were more likely to have BCR-ABL1 mutations compared to female patients, and the mean age was higher in patients with mutations (*p* < 0.05). There was a significant correlation between ABL KD mutations and gender (*p* = 0.004).

**TABLE 2:** Clinical, hematological and epidemiological characteristics of the patients at the time of diagnosis.

	Total (*n* = 163)	BCR-ABL mutation positive (*n* = 22)	*p*-value
Sex			
Male	80	17	<0.004^a^
Female	83	5	Chi = 8.08
Age, years			
Mean, 43 (range, 12–79)	163	53 (24–79)	0.231
≤49	86	9	Chi = 1.43
≥50	77	13	
Disease phase at screening			
CP	119	5	0.00001
AP	26	5	Chi = 53.12
BC	18	12	
Risk group			
Low	48	3	0.126
Mean	94	17	Chi = 4.13
High	21	2	
Spleen			
Normal	10	1	0.811
Medium (growth up to 8 cm)	118	17	Chi = 0.41
Massive (>8 cm)	35	4	
Hb			
Normal (120–160 g/L)	13	3	<0.480
Mild anemia (100–119 g/L)	49	4	Chi = 2.47
Mean anemia (80–99 g/L)	57	9	
Severe anemia (65–79 g/L)	42	6	
WBC			
Normal 3.9–9 (10^9^/L)	2	0	0.843
Leukopenia <3.9 (10^9^/L)	0	0	Chi = 0.34
Leukocytosis >9 (10^9^/L)	161	22	
PLT			
Normal 180–400 (10^9^/L)	92	12	0.920
Thrombocytopenia <180 (10^9^/L)	23	3	Chi = 0.16
Thrombocytosis >400 (10^9^/L)	46	7	
Blast cell (peripheral blood)			
0	62	3	0.017
1–10	99	18	Chi = 8.12
>10	2	1	
Eosinophil Basophil Association			
Eosinophils superiority	118	16	0.187
Basophilic superiority	20	4	Chi = 4.79
Without superiority	23	1	
No Association	2	1	
Additional chromosome			
Present	31	9	0.006
Absent	125	11	Chi = 10.13
Missing	7	2	
Blood group			
O (I)	49	6	0.645
A (II)	76	10	Chi = 1.66
B (III)	24	5	
AB (IV)	14	1	
Rh-factor			
Rh+	155	21	0.932
Rh−	8	1	Chi = 0.007
Seasons			
Spring	38	3	0.394
Summer	50	10	Chi = 2.98
Autumn	16	2	
Winter	57	6	
Missing	2	1	

CP, chronic phase; AP, acceleration phase; BC, blast crisis.

^a^ *p* < 0,05.

### Mutation analysis

**FIGURE 1:**
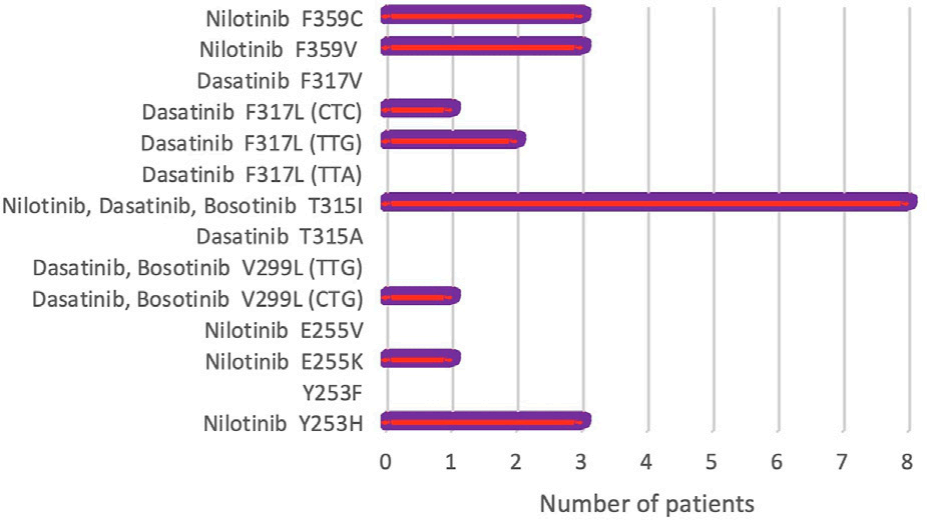
Frequency of rare BCR-ABL1 kinase domain mutations detected by Pyrosequencing in 163 CML patients. Mutations causing high-level resistance to second-line TKIs have been presented (NCCN, Version 2.2021) [28].

During a 5.5 years Pyrosequencing-based analysis (January 2014 to May 2019) of BCR-ABL1 kinase domain mutations, at least one point mutation was identified in 22 of 163 patients (13.4%). Figure 1 shows the frequency of the mutations, as well as mutations leading to high levels of resistance to second-line TKIs [28]. A total of 8 different point mutations were identified. Table 3 shows the frequency of identified mutations and their distribution by the disease phases. The most frequently identified mutation was T315I and mutations were mostly observed in patients in the BC or AP.

**TABLE 3:** Frequency of identified mutations and their distrubition according to the disease phases.

Mutations	CP	AP	BC	Total
T315I	—	1 (0.6%)	7 (4.4%)	8 (5.0%)
F359V	1 (0.6%)	1 (0.6%)	1 (0.6%)	3 (1.8%)
F359C	1 (0.6%)	1 (0.6%)	1 (0.6%)	3 (1.8%)
F317L (TTG)	1 (0.6%)	1 (0.6%)	—	2 (1.2%)
F317V (CTC)	1 (0.6%)	—	—	1 (0.6%)
Y253H	—	—	3 (1.8%)	3 (1.8%)
E255K	—	1 (0.6%)	—	1 (0.6%)
V299L (CTG)	1 (0.6%)	—	—	1 (0.6%)
Total	5 (3.0%)	5 (3.0%)	12 (7.4%)	22 (13.4%)
Total patient^a^	119 (73%)	26 (16%)	18 (11%)	163 (100%)

CP, chronic phase; AP, acceleration phase; BC, blast crisis.

More than 1 mutation was identified in 4 patients.

^a^ Total number of patients with and without mutations detected.

Additionally, only 1 mutation was identified in 18 (11%) patients, 2 mutations in 4 (2.4%) patients, and no mutation in 141 (86.6%) patients. Thus, 8 of 14 mutations were identified in 22 patients (13.4%) out of 163. The frequencies of the 8 mutations identified in our patient group were as follows: T315I—8 (36.4%), F359V—3 (13.7%), F359C—3 (13.7%), F317L (TTG)—2 (9.0%), F317V (CTC)—1 (4.5%), Y253H—3 (13.7%), E255K—1 (4.5%), V299L (CTG)—1 (4, 5%). The most common mutation appeared to be the T315I mutation followed by F359V, F359C, and Y253H (Table 3).

**FIGURE 2:**
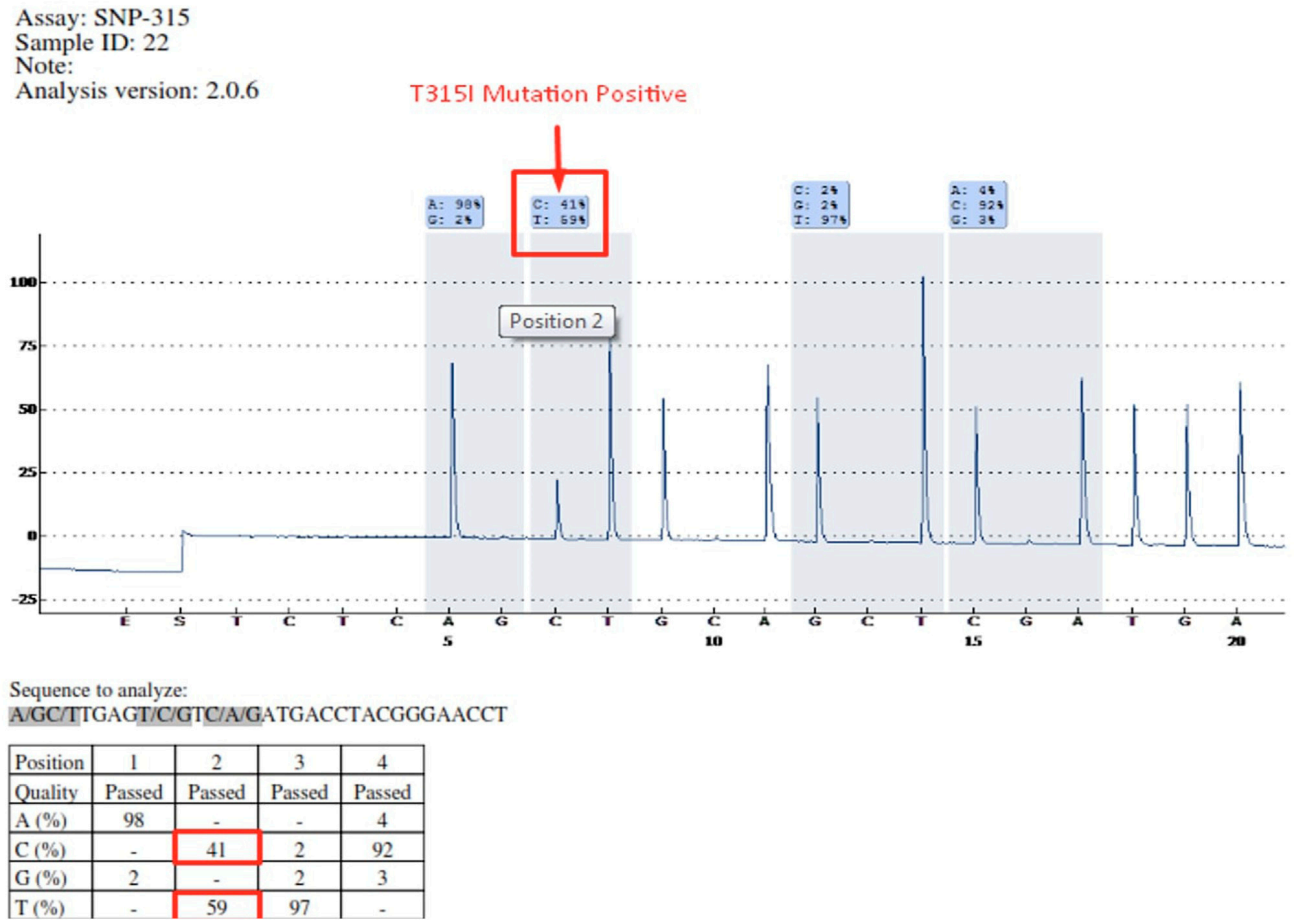
Determination of T315I mutation by Pyrosequencing method in patient 22 (C = 41%—T = 59%).

Mutations occurring in different regions of the ABL KD are presented in Table 4. Mutations occurring in the contact binding site region included T315I, F317L (TTA), F317L (TTG), F317L (CTC), F317V, T315A, which accounted for 50% of the mutations identified in the current study. The proportion of mutations identified in P-loop, SH2 domain and SH3 domain are presented in Table 4. Four patients had more than one mutation: F359V/F359C (3 patients), T315I/Y253H (1 patient). Figure 2 depicts the T315I mutation identified in the Contact binding site region of the ABL-kinase domain in patient 22 by Pyrosequencing method.

**TABLE 4:** The proportion of mutations occurring in distinct regions.

Region	Mutation	Proportion%
P-loop	E255K	18.1%
	E255V	
	Y253H Y253F	
Contact binding site	T315I	50%
	F317L (TTA) F317L (TTG) F317L (CTC) F317V	
	T315A	
SH2 domain	F359V	27.4%
	F359C	
SH3 domain	V299L (CTG) V299L (TTG)	4.5%

### Karyotyping

**FIGURE 3:**
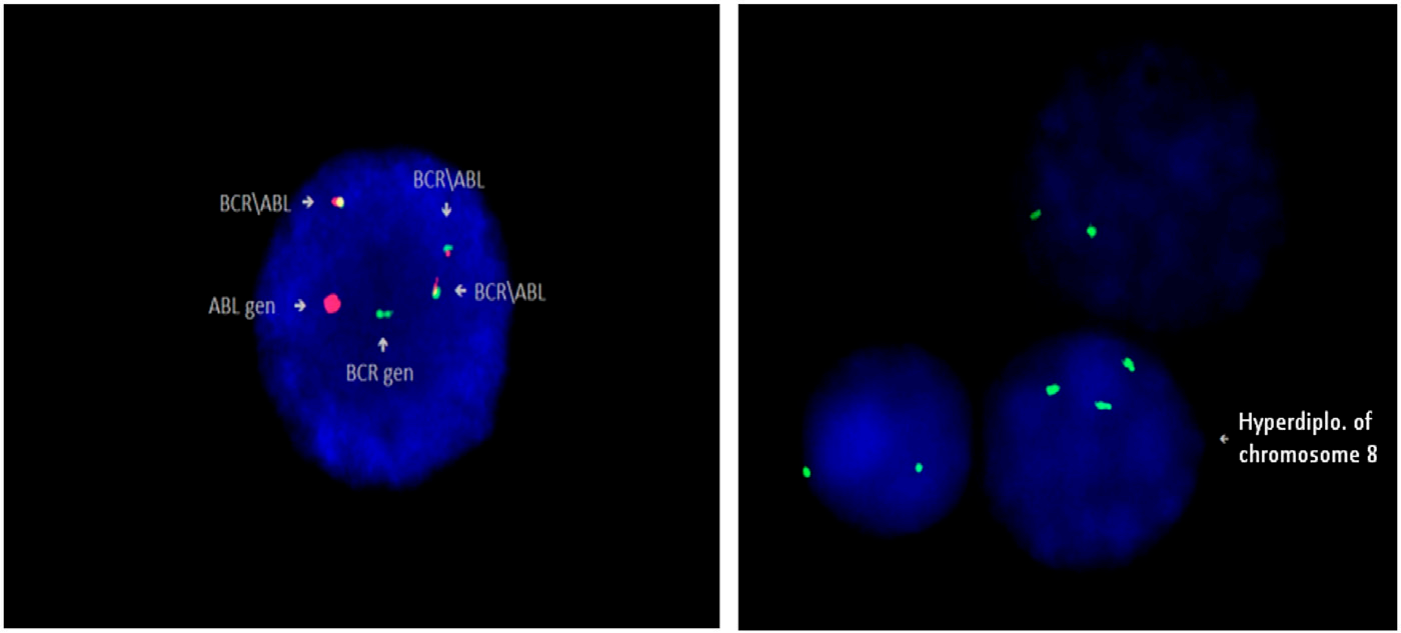
Identification of Philadelphia chromosome (Ph+) in patient 5, and trisomy 8 (+8) in patient 94 by FISH method (Cytocell).

Karyotype analysis was performed in 163 patients. It was not possible to identify the karyotype in 7 patients due the inability to obtain mitosis. Specimens for cytogenetic analysis were obtained during mutation screening of patients. Molecular cytogenetic (FISH) examination was performed in some samples in addition to cytogenetic analysis (Figure 3).

During the analysis, a duplication of the Philadelphia chromosome (+Ph), additional chromosomal aberrations and some structural aberrations were found in metaphases: (+8), (−7), (+19), (+21), i(17)(q10). Various chromosomal abnormalities were identified in 31 patients. ABL KD mutations were present in 9 out of 31 patients. Additional chromosomal aberrations were detected in 5 out of 8 patients with T315I mutations. Duplication of the Philadelphia chromosome (+Ph) was identified in 2 of them. We found an association between ABL KD mutations and additional chromosomal aberrations in CML patients (*p* = 0.006).

### Survival analysis

**FIGURE 4:**
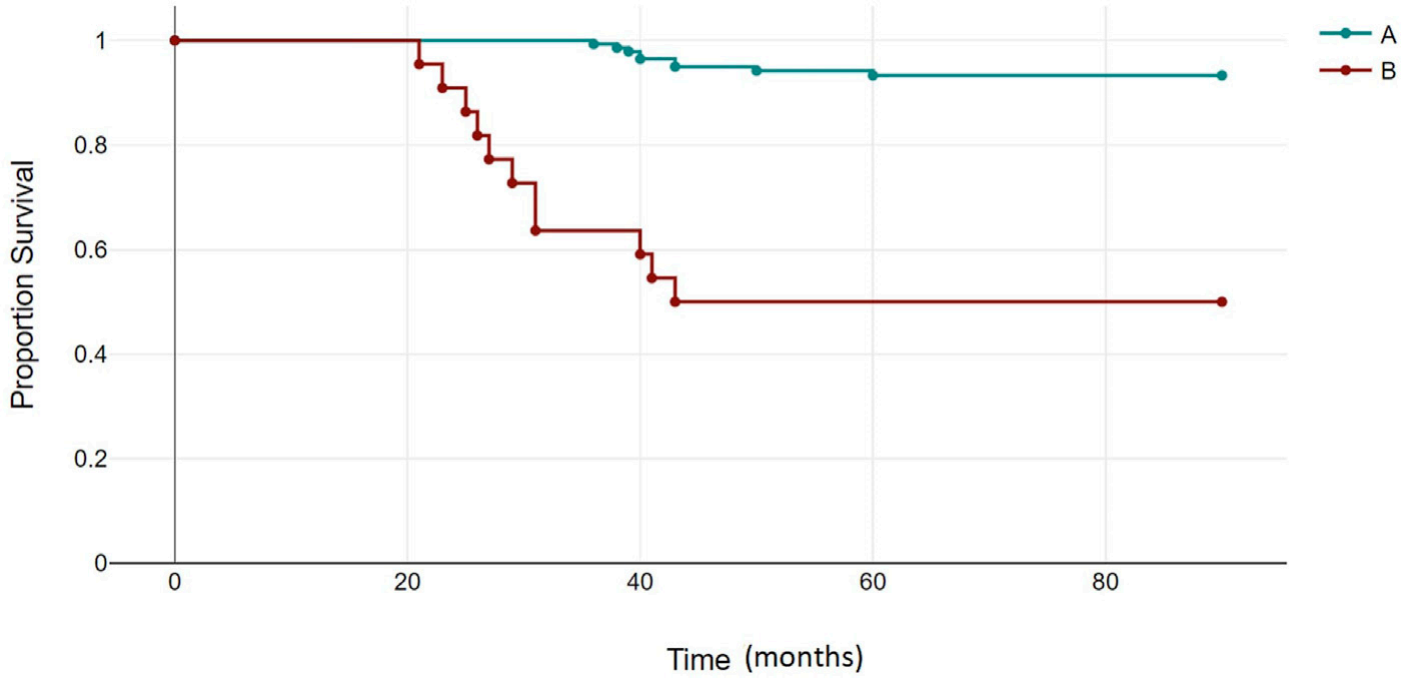
BCR-ABL1 negative (A) and BCR-ABL1 positive (B) OS based on mutational status of 163 CML patients. *p* < 0.00001.

**FIGURE 5:**
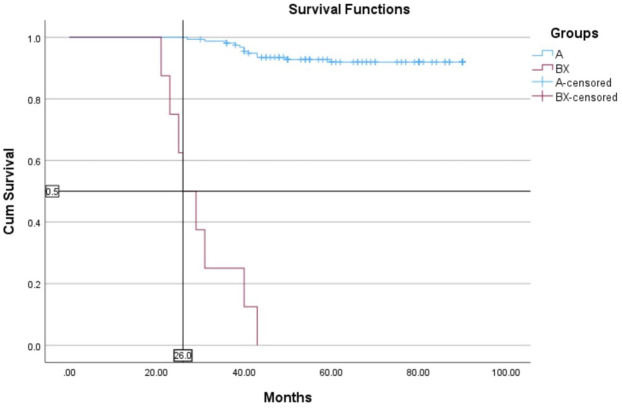
T315I negative (A) and T315I positive (BX) OS based on mutational status of 163 CML patients. *p* < 0.00001.

The median follow-up for all patients was 22 months (range, 2–89 months). The OS rates of patients in the BCR-ABL1 mutation-positive (B) and negative (A) groups were 50% and 93%, respectively (*p* < 0.00001) (Figure 4). Following 43 months, the OS indicators of the patient groups with T315I mutation (BX) and without (A) were 0% and 92%, respectively (*p* < 0.00001) (Figure 5). Estimated median overall survival for BX group was 26. However, for group A median overall survival was not reached at the time of analysis. These results indicated that the OS rate of patients with the T315I mutation was notably decreased.

## Discussion

Data from a cohort of 163 CML patients, subjected to mutation screening, were rigorously examined, revealing that 119 individuals (73%) were classified as being in the CP, 26 individuals (15.9%) in the AP, and 18 individuals (11.1%) in the BC. Remarkably, we identified 5 mutations in CP, 5 in AP, and 12 in BC. Our statistical analysis unveiled a pronounced association between mutations in the ABL kinase domain (ABL KD) and the respective disease phases, with a statistically significant *p*-value of <0.05.

Notably, the T315I mutation emerged as the predominant genetic aberration and exhibited a significant correlation with overall survival (OS). Patients afflicted with the T315I mutation (*n* = 7), T315I/Y253H (*n* = 1), Y253H (*n* = 2), and E255K (*n* = 1) mutations faced adverse clinical outcomes, culminating in mortality. By the end of the study, the majority of patients with T315I, Y253H and E255K mutations had progressed to the BC phase and they failed to respond to combination chemotherapy and second-line TKIs.

Our investigation conclusively established that these mutations were significantly associated with resistance to both first- and second-line TKIs. Within the cohort of patients in whom mutations were identified, four individuals demonstrated the presence of multiple mutations. Three patients exhibited co-occurrence of F359V/F359C mutations, while one patient manifested both T315I/Y253H mutations. However, due to the retrospective design of the study, it was not possible to confirm whether these mutations were complex or polyclonal. Furthermore, the limited representation of such patients in our study impeded the assessment of the potential impact of compound mutations on overall survival.

43-month survival was noted in 92% (142) of resistant patients without the T315I mutations. 30 (21%) out of 142 patients were treated with imatinib dose escalation, and the rest 112 (79%) patients received second-line TKIs. At the culmination of a 24 month interval, 48 patients (43%) subjected to second-line TKI therapy manifested MMR, while 64 individuals (57%) exhibited concurrent hematological response and achieved CCyR.

Subsequently, the cohort was stratified into low-, medium-, and high-risk groups based on their clinical characteristics at the time of initial CML diagnosis, revealing no significant associations between risk stratification, patients’ blood groups, Rh-factor, seasonality of morbidity, and ABL KD mutations. Additionally, the analysis failed to reveal any significant correlations between initial HB, WBC, PLT, eosinophil count, basophil count, spleen size at the time of diagnosis, and the presence of mutations. Nevertheless, a substantial association was observed between baseline blast cell count and the occurrence of ABL KD mutations, with a statistically significant *p*-value of <0.05.

The emergence of resistance to tyrosine kinase inhibitors (TKIs) can be dichotomized into BCR-ABL-dependent and BCR-ABL-independent processes. BCR-ABL-dependent mechanisms encompass BCR-ABL kinase overexpression and mutations within the BCR-ABL domain, while BCR-ABL-independent processes involve the activation of alternative signaling pathways, such as JAK/STAT, as well as the upregulation of efflux transporters or downregulation of influx transporters, resulting in reduced intracellular TKI levels [29]. Among these mechanisms, mutations in the BCR/ABL KD are of paramount importance. Intriguingly, our study affirmed that mutations in the KD, which confer resistance to TKI therapy, exhibit a markedly higher incidence in patients in the BC phase compared to those in the CP. Furthermore, patients with mutations in the CP category demonstrated an elevated propensity for progressing to AP or BC. Consequently, these mutations bear clinical significance by providing insights into patients who fail to achieve optimal responses to TKIs [30, 31]. Notably, the current therapeutic landscape offers a plethora of alternative options for TKI treatment contingent upon the specific mutations associated with TKI resistance [32, 33]. Acquired mutations, in particular, interfere with TKI binding or result in diminished TKI sensitivity [34]. Notably, distinct mutations display varying degrees of sensitivity to TKIs, underscoring the importance of mutation detection in guiding TKI selection and influencing patient prognosis. These principles align with recommendations from both the European LeukemiaNet (ELN) and the National Comprehensive Cancer Network (NCCN), advocating the consideration of mutational profiles in the determination of subsequent therapy [13, 35].

It is noteworthy that, owing to the contribution of TKIs and an enhanced understanding of the influence of the BCR-ABL mutational profile on drug efficacy, CML is presently recognized as a chronic ailment. However, it remains pertinent to acknowledge that a subset of patients continues to experience disease progression. Consequently, there is an imperative need for further investigations elucidating the intricate interplay between genes and drugs, with the aim of achieving optimal coverage in the treatment of CML.

## Data Availability

The raw data supporting the conclusions of this article will be made available by the authors, without undue reservation.
